# MCE-HGCN: Heterogeneous Graph Convolution Network for Analog IC Matching Constraints Extraction

**DOI:** 10.3390/mi16060677

**Published:** 2025-06-03

**Authors:** Yong Zhang, Yong Yin, Ning Xu, Bowen Jia

**Affiliations:** 1 School of Information Engineering, Wuhan University of Technology, Wuhan 430070, China; yongz@whut.edu.cn (Y.Z.); yiyng_hust@126.com (Y.Y.); 2 Chongqing Research Institute, Wuhan University of Technology, Chongqing 401151, China

**Keywords:** analog IC, matching constraints, heterogeneous multi-graph, mixed attentions, small dataset

## Abstract

Matching constraints in an analog integrated circuit (IC) are critical to optimizing layout performance. To extract these matching constraints accurately and efficiently from the netlist, we propose the heterogeneous matching constraint extraction graph neural network (MCE-HGCN). First, the netlist is mapped into a heterogeneous attribute multi-graph, and based on the characteristics of analog IC matching constraints, a mixed-domain attention mechanism is developed to leverage both the topology information and node attributes in the graph to characterize node embeddings. A matching classifier, implemented using the support vector machine (SVM), is then employed to classify different types of matching constraints from the netlist. Additionally, a matching filter is introduced to remove interference terms. Experimental results demonstrate that the MCE-HGCN model converges effectively with small datasets. In the matching prediction process, the mean *F*1 score reached 0.917 across different netlist processes and circuit types while maintaining a shorter runtime compared to other methods. Ablation experiments also show that incorporating the mixed-domain attention mechanism and the matching filter individually leads to significant performance improvements. Overall, MCE-HGCN excels at extracting matching constraints from various analog circuits and processes, offering valuable insights for placement guidance and enhancing the efficiency of analog IC layout design.

## 1. Introduction

To mitigate the influence of factors such as parasitic effects and process variations on the performance of analog IC chips, various topological constraints must be incorporated into the layout design [1]. These constraints typically include symmetry, matching, and proximity requirements. Symmetry constraints ensure that devices are placed in mirror-image locations. Matching constraints mandate either axisymmetric placement or placement that balances the center of mass across the device layouts. Proximity constraints dictate that devices be positioned in specific locations, such as within a shared substrate, a common trap region, or enclosed by a guard ring, often in alignment with their matched counterparts [2]. Mismatches between sensitive devices in critical circuit blocks can significantly degrade overall performance. For instance, mismatches in differential circuits can disrupt current balance and reduce the circuit’s common-mode rejection ratio (CMRR). To enhance circuit robustness and achieve optimal performance metrics, it is essential to carefully consider and implement various matching constraints during the layout design process.

Traditionally, the identification of matching constraints in analog IC design relies on the expertise of seasoned designers. This manual process is not only time-intensive but also subjective, as it varies with individual judgment. Classical approaches to automating the extraction of matching constraints are typically based on sensitivity analysis. However, these methods struggle to scale effectively for large and complex analog ICs [3]. Alternatively, data mining techniques infer constraints in new designs by comparing them with labeled knowledge bases. However, the intricate nature of analog IC design poses significant challenges to constructing comprehensive design pattern libraries [4,5]. Recently, graph neural networks (GNNs) [6] have shown promise in aiding analog IC design, with applications such as parasitic prediction, layout performance evaluation, and layout decomposition. In the realm of constraint extraction, Gao et al. [7] introduced a graph learning framework that transforms symmetry constraint detection into a binary classification problem. They employed rule-based and probability-based filters to eliminate misclassifications and enhance detection accuracy. Similarly, Kunal et al. [8] used a graph convolutional network to traverse circuit graphs, embedding node features and domain-specific information into vectors. By applying a two-layer fully connected neural network, they estimated graph edit distances (GED) to identify symmetry constraints. However, these supervised learning approaches require extensive labeled datasets. To address symmetry constraint extraction at different levels of analog IC modules, Kunal et al. [9,10] represented circuit netlists as undirected bipartite graphs, created hierarchical representations, and automatically identified sub-circuit modules to extract constraints across module levels. Other methods, such as the approach in [11], analyze signal flow and use graph automorphism to detect graph symmetries, while Eick et al. [12] generate constraints directly through pattern matching and organize them hierarchically using structural signal flow graphs. Kunal et al. [13] attempts to integrate GNNs with graph-based algorithms to achieve layout constraint extraction at different design levels. Unlike the aforementioned method that directly extracts constraints from SPICE netlists, Yao et al. [14] proposed a layout-based symmetry constraint extraction method. However, this approach requires a large number of high-quality layouts.

For matching constraint extraction, Liu et al. [15] applied a two-sample Kolmogorov–Smirnov (K–S) test [16] and heuristic algorithms to assess graph similarity in system-level circuits. Their scalable graph similarity algorithm, based on spectral analysis, identifies matched device pairs, though its applicability is often hindered by the computational complexity of the statistical analysis. Chen et al. [17] proposed an unsupervised learning framework for symmetry constraint extraction using GNNs. By representing circuit netlists as heterogeneous graphs, they leveraged unsupervised GNN embeddings to derive device-level matching constraints. Sub-circuit feature embeddings were further obtained through neighbor sampling and aggregation, while cosine similarity calculations identified circuit-level symmetry relations among candidate pairs. In short, most research on analog IC constraint extraction has predominantly focused on identifying symmetry constraints or locating matching device pairs. Furthermore, Xu et al. [18] proposed a netlist information extraction method based on the edge-augmented graph attention network (EGAT) to fully leverage the connectivity characteristics of circuits and device information for learning the general rules of symmetry constraints. However, these efforts of-ten fail to discern the specific structures of matching constraints, offering limited practical guidance for placement tasks [19].

In this paper, we construct the netlist as a heterogeneous attribute multigraph and enhance a graph convolutional network with mixed-domain attention mechanisms on nodes and edges. This approach enables the training of node embedding models and the prediction of node embeddings within the graph. By calculating the Euclidean distances between node pairs and integrating manually labeled matching information, the analog IC matching constraint extraction problem is reformulated as a triple classification task using a support vector machine (SVM). During the matching prediction process, a matching classifier identifies matching types in new netlists, while a matching filter eliminates non-adjacent matching pairs to mitigate the impact of unrelated node embeddings. This process reduces misclassification and provides more accurate guidance for layout generation. Our primary contributions are as follows:

(1) Framework for matching constraint extraction: We propose a framework based on heterogeneous graph convolutional networks to identify matching structure types required for layout placement from analog netlists. The matching constraint extraction task is reformulated as a classification problem involving node pairs within a heterogeneous graph.
(2) Mixed-domain attention mechanism: We enhance the heterogeneous graph neural network with a mixed-domain attention mechanism that considers both nodes and edges. This improvement facilitates more effective message passing between different node and edge types, maximizes the utility of small datasets, and strengthens the identification of matching structures in netlists.
(3) Matching classifier and filter: A matching classifier based on support vector machines is proposed to identify matching structures within circuits. Additionally, matching filters are implemented to further improve recognition accuracy.
(4) Experimental validation: Experimental results confirm the effectiveness of the proposed method in extracting matching constraints across various ICs and processes. This approach provides layout engineers with valuable support in determining device matching relationships.

The rest of the paper is organized as follows: Section 2 introduces the problem description of matching constraint extraction and the method for constructing the graph. Section 3 describes the proposed heterogeneous graph neural network framework MCE-HGCN for extracting matching constraints in analog IC. Section 4 represents the experiments and results. Finally, Section 5 concludes the paper.

## 2. Problem Description and Graph Construction

### 2.1. Problem Description

**Figure 1:**
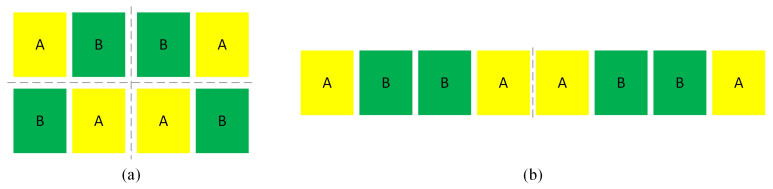
Matching layout. (**a**) Common centroid matching layout; (**b**) axisymmetric matching layout.

Matching constraints in analog ICs are often implemented to ensure a consistent gate orientation between devices, thereby reducing performance mismatches caused by processing variations and enhancing device symmetry [20,21]. Common matching layouts, are shown in Figure 1, are typically categorized into common centroid and axisymmetric structures. In Figure 1a, two MOS devices are divided into eight smaller segments, arranged crosswise to share the same center of mass. This structure is commonly used in circuits requiring high precision matching, such as differential pairs. By mitigating offsets introduced by fabrication process errors, this layout improves circuit performance and stability. In Figure 1b, on the other hand, eight smaller MOS devices are symmetrically cross-placed. This configuration enhances the module’s symmetry, reduces common-mode noise interference, and increases the common-mode rejection ratio (CMRR) of the circuit. It is frequently employed in high current mirror circuits to maintain consistent current flow direction.

### 2.2. Heterogeneous Attribute Multi-Graph

Analog IC can be naturally represented as graph data due to the structural similarity between their topologies and graph data structures. In this study, the analog IC netlist is modeled as a heterogeneous attribute multigraph, $G=\left(V,E,A,R\right)$, where each device in the netlist is denoted as a node, v∈V, and V represents the set of all types of nodes. A directed edge, $e=\left(u,v,\tau_{v}\right)\in E$, denotes the interconnection of an edge of type $\tau_{v}$ from a vertex u to a vertex v, where the edge type is denoted by the pin type of the node v that is connected to it by e, E is the set of all types of directed edges, and A and R denote the associated sets of node types and the set of edge types, respectively.

**Figure 2:**
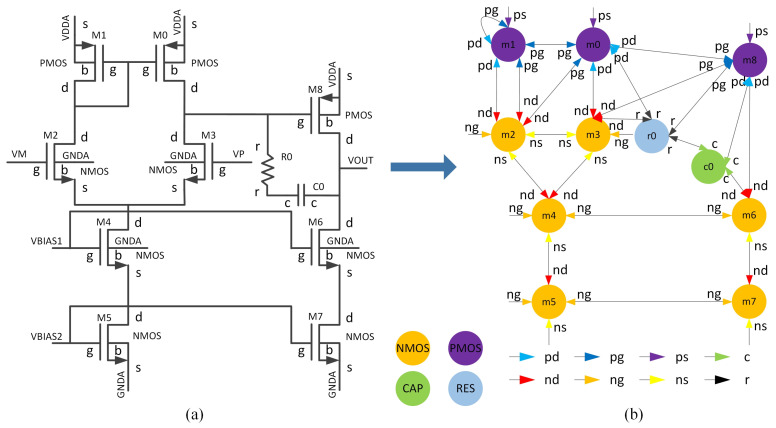
Heterogeneous attribute multi-graph construction process: (**a**) analog IC schematic; (**b**) heterogeneous attribute multi-graph.

Figure 2 illustrates the construction process for the heterogeneous attribute multigraph of an analog IC. On the left is the circuit schematic corresponding to the netlist, and on the right is the resulting heterogeneous attribute graph. The schematic consists of three PMOS (m0, m1, m8), six NMOS (m2, m3, m4, m5, m6, m7), a capacitor (c0), and a resistor (r0). These components are mapped to four distinct node types: PMOS, NMOS, CAP, and RES. The wire nets in the schematic are represented as directed edges in the graph, with edge types defined by the pin types of the connected nodes. These pin types include nd, ng, ns (drain, gate, and source of NMOS), pd, pg, ps (drain, gate, and source of PMOS), and c, r (pins connected to the capacitor and resistor).

## 3. MCE-HGCN Framework

### 3.1. MCE-HGCN Network

**Figure 3:**
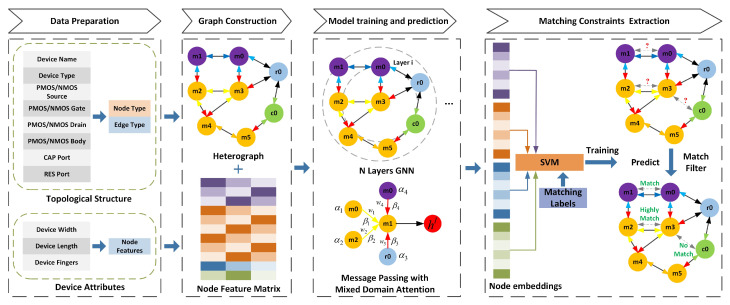
Computation flow of the proposed MCE-HGCN framework.

Existing graph neural networks face limitations in effectively extracting matching constraints. To address this, the MCE-HGCN network is proposed to enhance the aggregation of attribute features for different node and edge connections in heterogeneous attribute multigraphs [22,23]. Figure 3 illustrates the workflow of MCE-HGCN, which consists of four main stages: data preparation, graph construction, model training and prediction, and matching constraint extraction. First, in the data preparation and graph construction stages, the connection network between devices in the netlist and their respective attributes are represented as a heterogeneous graph and a node feature matrix.

The message passing computation procedure for the traditional inductive graph convolution operation [24] is described in Equation (1). The embedding representation $h_{u}^{l}$ at layer l is calculated by aggregating information from the node embeddings at the previous layer (l−1) and their neighboring nodes.

(1) $$h_{u}^{l}=\sigma (W\times Agg(h_{u}^{l-1},\sum_{v\in N_{u}}h_{v}^{l-1})),$$

where $\sigma \left(\times \right)$ is the activation function, W is the learnable weight matrix, and Agg is the aggregation function. The commonly used aggregation functions are the average aggregation function, the long short-term memory (LSTM) aggregation function, the pooling aggregation function, etc. $h_{u}^{l-1}$ is the embedding representation of the node at the layer *l* − 1, and *v* is the neighboring node of the node *u* belonging to the set of neighboring nodes $N_{v}$.

### 3.2. Mixed-Domain Attention Mechanism

In heterogeneous graphs, message passing between nodes of the same type has been emphasized in numerous studies due to the stronger influence and correlation observed within such nodes. However, this approach often overlooks the contributions of nodes and edges of different types in the local neighborhood, which are critical for capturing the topological information in analog IC netlists. To address this limitation, a mixed-domain attention mechanism is proposed, incorporating node attention and edge attention [25,26]. This mechanism enables the model to better capture the interplay between different types of neighboring nodes and edges, ensuring effective message passing.

#### 3.2.1. Node Attention

Node attention captures the importance of neighboring nodes of different types while reducing the impact of noisy nodes. Take a node u of type t and its *N* neighbor nodes $v_{i}\in N_{u}$ of type $t_{i}\in A$, where the embedding of node u is denoted as $h_{u}=\sum_{v_{i}\in N_{u}}{\tilde{A}}_{u,v}\times h_{v}$. In the analog IC matching constraint extraction task, matching constraints typically exist between devices of the same type but not across different types. To reflect this, the learning parameter vectors μ for neighboring nodes of different types are set to 0 during the message passing, resulting in an attention score of 0. The attention score $a_{v_{i}}$ of neighboring node $v_{i}$ to node u is as fellow:

(2) $$a_{v_{i}}=\left\{\begin{array}{c} 0,t_{i}\neq t\\ \sigma \left(\mu^{T}\left[Wh_{u}||Wh_{v_{i}}\right]\right),t_{i}=t \end{array}\right.,$$

where $h_{u}$ and $h_{v_{i}}$ are the feature vectors of node u and node $v_{i}$, respectively, and || denotes the splicing operation. Equation (2) is normalized by the SoftMax function to obtain the attention weights of the message passing neighbor nodes, as shown in Equation (3).

(3) $$\alpha_{u,v_{i}}=softmax\left(a_{v_{i}}\right).$$

#### 3.2.2. Edge Attention

**Figure 4:**
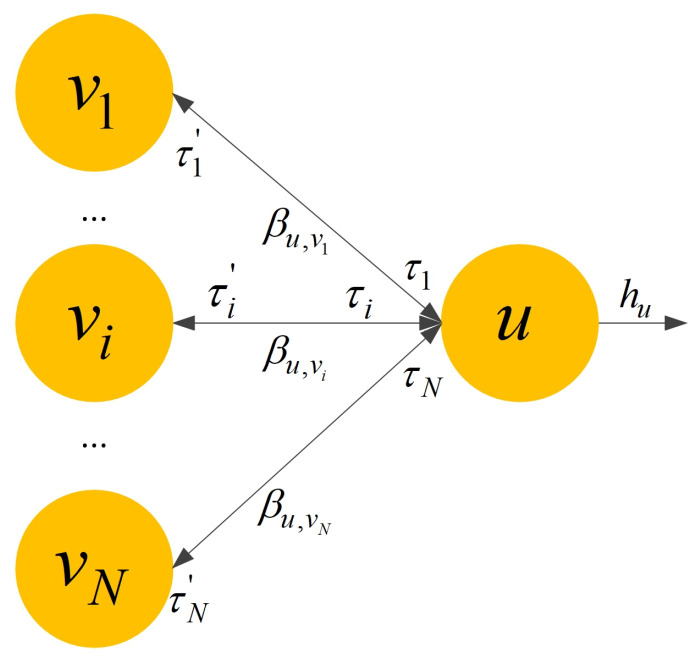
Edge attention mechanism.

Edge attention distinguishes the influence of different edge types on the embedded information of nodes during message passing. Take a node u with type t and its *N* neighbor nodes, $v_{i}\in N_{u}$, the edge type of node $v_{i}$ connecting to node u is $\tau_{i}\in R$, and the edge type of node u connecting to node $v_{i}$ is $\tau_{i}^{\prime }\in R$. The edge attention mechanism is shown in Figure 4. In the analog IC matching constraint extraction, the possibility of matching between devices is higher if there is a co-polar connection between devices (i.e., two graph nodes are interconnected with the same edge type), so the message passing attention score $b_{v_{i}}$ for edge type $\tau_{i}$ between neighboring nodes is computed, as shown in Equation (4).

(4) $$b_{v_{i}}=\left\{\begin{array}{c} \sigma \left({\rho \gamma }^{T}\left[Wh_{u}\right]\right),\tau_{i}\neq \tau_{i}^{\prime }\\ \sigma \left(\gamma^{T}\left[Wh_{u}||Wh_{v_{i}}\right]\right),\tau_{i}=\tau_{i}^{\prime } \end{array}\right..$$

The attention weights of the message passing edges are obtained by normalization using the *softmax* function, as shown in Equation (5).

(5) $$\beta_{u,v_{i}}=softmax\left(b_{v_{i}}\right).$$

Given that neighboring nodes may have distinct features and edge types, a simple concatenation operation may not effectively capture their relationships. To better encode matching features in node embeddings, we adopt a multiplication-based blending operation. The heterogeneous graph convolution operation, combining node and edge attention, is formulated as follows:

(6) $$h_{u}^{l}=\sigma (W\times Agg(h_{u}^{l-1},\sum_{v_{i}\in N_{u}}{\alpha_{u,v_{i}}\times \beta_{u,v_{i}}\times h}_{v}^{l-1})).$$

This mixed-domain attention mechanism regulates the message passing between different nodes and edge types. We enhance the message passing between matching nodes and reduce the interference of other nodes’ information on the prediction of matching results. The enhanced graph neural network has three layers. The model input dimensions are *n* × 3, where *n* is the number of nodes in each heterogeneous graph. The network uses the ReLU6 activation function, and its hidden layers contain 16 neurons.

### 3.3. Matching Constraint Extraction Methods

#### 3.3.1. Matched Classifier

Based on the constructed heterogeneous graph neural network model, the embedding representation of each node in the heterogeneous graph is computed, and the matching constraint value between two nodes is determined using Euclidean distance [27], as defined in Equation (7).

(7) $$d\left(u,v\right)=\sqrt{\sum_{i=1}^{n}{\left(h_{u}^{i}-h_{v}^{i}\right)}^{2}},$$

where $d\left(u,v\right)$ represents the Euclidean distance between nodes u and v; $h_{u}^{i}$ and $h_{v}^{i}$ are the values of the embedding vectors for u and v in the *i*-th dimension, respectively; and *n* is the dimensionality of the embedding vectors. Using this distance, combined with matching information extracted from the layout, the matching relationships between nodes are categorized as no match, match, or highly match, corresponding to independent devices, axisymmetric matching structures, and common centroid matching structures, respectively. In analog ICs, the relationships between nodes (devices) are complex, often involving multiple edges. This complexity makes identifying the type of node pairs challenging when a graph neural network serves as the output layer. To address this, we train a matching classifier using a support vector machine (SVM) to predict the matching relationships in new netlists, improving the efficiency of layout design. The implementation is detailed in lines 4–8 of Algorithm 1, where node pairs in the graph data are sequentially identified, their Euclidean distances are calculated, and these distances are combined with labeled information from the corresponding netlist to train the classifier.
**Algorithm 1** Matchings Predict Model Training**Input:** Heterogeneous attribute multi-graph datasets $G_{s}$, Annotated matching relationship $P=\left\{1:\left[m_{1},m_{2},\cdots ,m_{i}\right],\cdots ,n:\left[m_{1},m_{2},\cdots ,m_{j}\right]\right\}$.
**Output:** Matching predict model M.
1. Initialize the MCE-HGCN net;
2. **for each** graph g in $G_{s}$
**do**
3.  predict graph embedding $H_{g}$;
4.  **for each** node i in graph g
**do**
5.      **for each** node i+1 in graph g
**do**
6.        Compute the Euclidean distance d between node i and node i+1 by nodes’ embedding;
7.        Add d to Euclidean distance set D between node pairs; $D=\left\{d_{1},d_{2},\cdots ,d_{N}\right\}$, N is the number of nodes in the graph;
8.  Support vector machine training SVM(D, P); 9. **return**
M.

#### 3.3.2. Matched Filter

During the message passing process, the embedding values of non-adjacent nodes may sometimes appear similar, potentially introducing noise into the prediction of matching constraints. To mitigate this issue, we design a matching filter. After the matching classifier predicts the matching pairs, the filter uses the neighbor relationships in the graph to eliminate non-adjacent node pairs from the results.

The specific implementation of the matching filter is outlined in lines 4–6 of Algorithm 2. For a predicted matching pair $p=\left(u,v\right)$, where p∈P and $G_{P}=\left(S,D\right)$ represents the graph’s node pair set (with S as the list of source nodes and D as the list of target nodes), adjacency is determined as follows: if $p\in G_{P}$, then node u and v have adjacency; otherwise, they are non-adjacent node pairs and are removed from the prediction result. This filtering step ensures that only relevant node pairs are considered in the matching predictions, thereby improving the accuracy and reliability of the constraint extraction process.
**Algorithm 2** Matching Constraints Extraction**Input:** An analog circuit netlist $n_{i}$. **Output:** Matching pairs $P_{s}$ in the netlist.
1. Construct the heterogeneous attribute multi-graph
$g_{i}$ form the netlist
$n_{i}$;
2. MCE-HGCN predicts nodes’ embedding
$H_{g_{i}}$ in
$g_{i}$;
3. Predicting matching node pairs p by
$SVM\left(H_{g_{i}}\right)$;
4. **for each** node pair p∈P
**do**
5.  **if** $p\in G_{P}$
**then**
6.     Add p to $P_{s}$; 7. **return** $P_{s}$.

## 4. Experiment

### 4.1. Experimental Data

**Figure 5:**
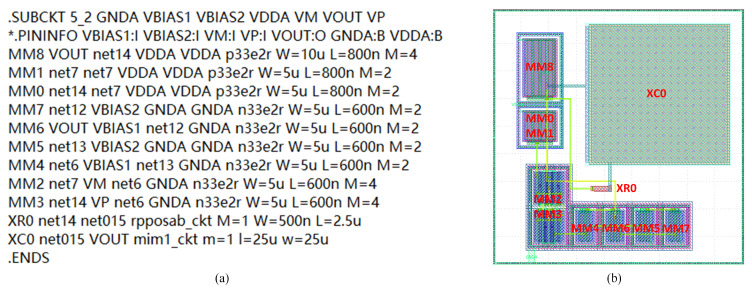
OTA circuit netlist and layout. (**a**) OTA circuit netlist; (**b**) OTA circuit layout.

The experimental dataset includes netlists and manual layouts for operational transconductance amplifier (OTA) circuits and low dropout regulator (LDO) circuits, using SMIC 130 nm and 180 nm processes [28]. Additionally, the dataset contains data for comparators (COMP) and analog-to-digital converters (ADC) from the TSMC 40 nm process, sourced from the open-source MAGICAL layout framework. Inter-device matching constraints in the netlist are labeled based on the actual matching relationships and types observed in the corresponding layout. As illustrated in Figure 5, PMOS devices MM0 and MM1 exhibit axisymmetric placement and are labeled as “match”. NMOS devices MM2 and MM3 follow a common centroid placement and are labeled as “highly match”. Devices MM4, MM5, MM6, MM7, MM8, XR0, and XC0 are independent devices and are labeled as “no match”.

The specific data distribution is shown in Table 1. The heterogeneous attribute multi-graph dataset is constructed by Algorithm 1, which creates the relationship graphs for nodes and edges, while also labeling the matching device pairs in each graph.

### 4.2. Model Training

The experiments were conducted using the Windows 10 64-bit operating system on a computer configured with an Inter(R) Core (TM) i5-13600KF 3.5 GHz and an NVIDIA GeForce GTX 4060 Ti 16 G graphics card. The model training, validation, and testing were carried out using the PyTorch 1.7.1 deep learning framework and the DGL 0.7.2 graph neural network learning framework. The model training process was set to run for 1000 epochs, with the Stochastic Gradient Descent (SGD) optimizer. The learning rate was configured to 0.0001, and the cross-entropy loss function was used. The regularization parameter for the SVM classifier was set to 1, with a linear kernel function and a one-vs-rest multi-class strategy.

**Figure 6:**
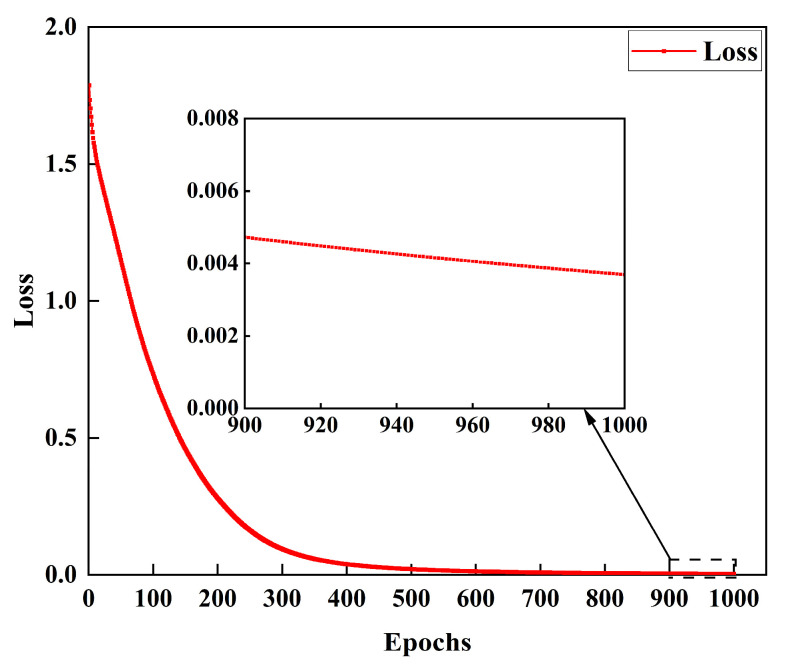
MCE-HGCN model training loss curve.

The training loss curve for the MCE-HGCN model is shown in Figure 6. After approximately 1000 epochs, the network loss stabilized at around 0.003, indicating that the model had converged and various metrics had become stable.

### 4.3. Experimental Analysis

#### 4.3.1. Comparison Experiments

The experiments compare the proposed method with pattern matching [12], unsupervised learning [18], and the inductive graph neural network GraphSAGE [22] trained with supervised learning. The evaluation metrics for prediction include accuracy, *F*1 score, precision, and recall. Accuracy measures the overall correctness of the predictions, while the *F*1 score is especially useful for evaluating classification results in datasets with imbalanced categories (e.g., matched vs. non-matched). It provides a more comprehensive measure of the model’s classification performance. We implemented pattern matching and unsupervised learning methods using open-source code and the collected data. We modified their outputs to include accuracy Acc, *F*1 score F1−score, and prediction time. The formulas for Acc and F1−score are as follows:

(8) $$Acc=\frac{TP+TN}{TP+FN+FP+TN},$$

(9) $$F1-score=\frac{2\times P\times R}{P+R},$$

where precision $P=\frac{TP}{TP+FP}$, recall $R=\frac{TP}{TP+FN}$, and TP, FP, TN, and FN represent the number of true positive predictions, false positive predictions, true negative predictions, and false negative predictions, respectively.

The experimental results are summarized in Table 2, which includes the matching constraint prediction time, accuracy, *F*1 score, precision, and recall for OTA netlists (130 nm and 180 nm processes), LDO netlists (130 nm process), COMP netlist (40 nm process), and ADC netlists (40 nm process). Clearly, the proposed MCE-HGCN outperforms the pattern matching, unsupervised learning, GraphSAGE, and EGAT methods across all five test sets, achieving higher prediction accuracy and *F*1 score. The average prediction accuracy reaches 98.2%, indicating high precision, while the average *F*1 score of 0.917 demonstrates the model’s strong ability to predict matching constraints in the test netlists. These results are further supported by the precision and recall metrics. Although the prediction time for OTA netlists is slightly longer than that of the pattern matching and GraphSAGE methods, the difference is minimal, with an average prediction time of only 0.80 s. In the future, we will also apply MCE-HGCN to more advanced technologies and different types of circuits to extract matching constraints from their netlists.

**Figure 7:**
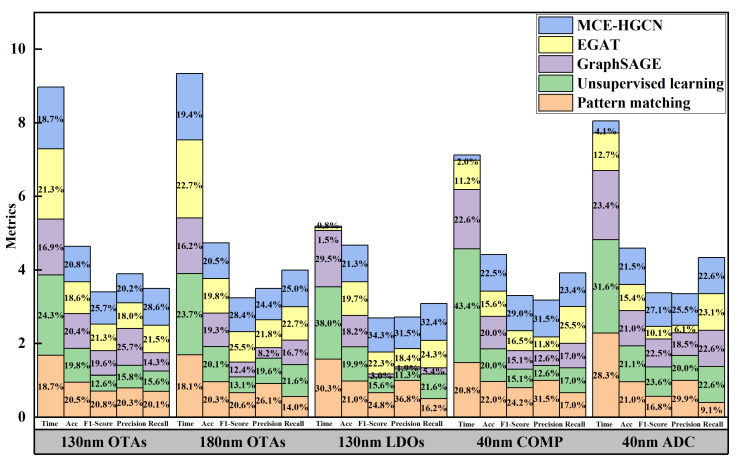
Stacked histograms of matching constraint extraction results from different methods. The percentages in the figure are rounded to one decimal place.

To enable a clearer comparison of experimental outcomes, we employed stacked histograms illustrating the proportion of each metric (accuracy, *F*1 score, precision, and recall) for every test case across different methods. Each metric’s proportion was calculated by dividing its value by the total across all five methods. As shown in Figure 7, the proposed MCE-HGCN approach consistently outperformed other methods, achieving the highest accuracy and *F*1 score proportions for OTA circuits under both 130 nm and 180 nm processes, all within a comparable prediction time frame. Furthermore, for the 130 nm LDO, 40 nm COMP, and ADC circuits, MCE-HGCN required only 0.8%, 2.0%, and 4.1% of the total prediction time frame, while still delivering the highest *F*1 score proportion among the methods evaluated. These results indicate MCE-HGCN’s efficiency and adaptability in extracting matching constraints across varied circuits types and processes, offering valuable support for layout design engineers.

**Figure 8:**
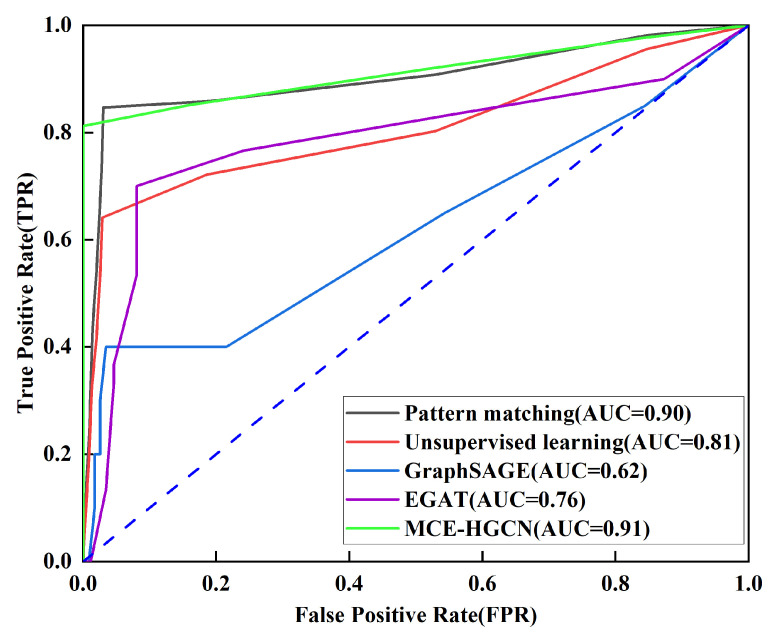
Micro-average ROC curves of different methods.

The micro-average receiver operating characteristic (ROC) curve for the experimental tests is shown in Figure 8, illustrating the testing curves and area under the curve (AUC) values for pattern matching, unsupervised learning, GraphSAGE, EGAT, and the proposed approach. The ROC curve for MCE-HGCN is closest to the upper-left corner, indicating that the model maintains a low false-positive rate (FPR) and a high true-positive rate (TPR) across various thresholds. The AUC value of 0.91 is the highest, suggesting excellent performance in identifying matching constraints in the netlist.

#### 4.3.2. Ablation Experiment

**Figure 9:**
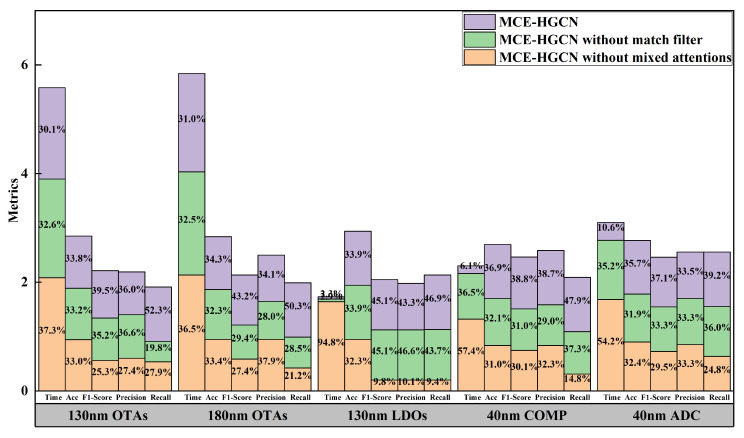
Stacked histogram of matching constraint extraction with different optimization factors. The percentages in the figure are rounded to one decimal place.

To validate the optimization effects of this work on the original network, a series of ablation experiments were conducted. These experiments focused on assessing the impact of the mixed-domain attention mechanism on match extraction and the performance of match filters. The experimental results are shown in Table 3 and Figure 9. According to Table 3, when the mixed-domain attention mechanism is omitted, the model achieves higher prediction accuracy but generally lower *F*1 score, leading to poorer predictive performance for match types. On the other hand, when the match filter is not included, interference from non-connected components affects the prediction results, resulting in reduced prediction accuracy and *F*1 score. Figure 9 illustrates the distribution of validation metrics across five test cases, comparing scenarios with and without the mixed-domain attention mechanism and matching filter. The results clearly indicate that incorporating these two components significantly enhances performance. Specifically, MCE-HGCN showed consistent improvements in accuracy, *F*1 score, precision, and recall, while maintaining a similar or reduced time proportion compared to approaches lacking these components. This confirms the effectiveness of the mixed-domain attention mechanism and matching filter in optimizing matching constraint extraction

## 5. Conclusions

In this paper, we proposed an analog IC matching constraint extraction method based on graph convolutional neural networks (GCNs). First, a heterogeneous attribute multi-graph dataset was constructed using the interconnection relationships and device attributes from the input netlist. Next, to effectively capture the characteristics of matching devices, we developed a mix-domain attention mechanism that operates at both node and edge levels. This mechanism manages the exchange of information between matching devices, significantly improving the similarity of their embedding representation. A support vector machine (SVM) was employed as the matching classifier to categorize different types of matching constraints. Additionally, a matching filter was designed to reduce the influence of non-interconnected devices on the prediction outcomes.

The effectiveness of this method was demonstrated through comparisons with traditional pattern matching, unsupervised learning, and the GraphSAGE, and EGAT methods, as well as through ablation experiments. The following conclusions were drawn:

(1) For small datasets, leveraging the characteristics of inter-device relationships through attention mechanisms significantly optimized the message passing in the graph neural network, enabling effective prediction of matching constraints between devices; (2) the introduction of matching filters successfully mitigated interference from non-connected devices, further enhancing the accuracy and robustness of the matching constraint extraction process; and (3) the MCE-HGCN effectively extracted matching constraints from the netlist, assisting layout engineers in quickly determining the spatial arrangement of devices. This, in turn, improved the efficiency of analog layout design and provided valuable insights for intelligent analog layout generation, guiding the placement of devices and enhancing the overall design performance.

## Figures and Tables

**Table 1:** Statistics of the experimental dataset.

Circuit Type	Number of Training Dataset				Number of Test Dataset			
	Circuits	Unmatched Pairs	Matched Pairs	Highly Matched Pairs	Circuits	Unmatched Pairs	Matched Pairs	Highly Matched Pairs
130 nm OTAs	100	7914	407	92	8	265	20	8
130 nm LDOs	4	588	18	6	1	147	6	0
180 nm OTAs	43	1901	127	51	5	321	21	5
40 nm COMP	/	/	/	/	1	125	8	3
40 nm ADC	/	/	/	/	1	1464	77	19

**Table 2:** Matching constraint extraction results for different methods.

Metrics		130 nm OTAs	180 nm OTAs	130 nm LDOs	40 nm COMP	40 nm ADC	Average
Pattern matching [12]	Time/s	1.68	1.69	1.57	1.48	2.28	1.74
	Acc/%	94.9	96.3	98.0	97.1	96.3	96.5
	F1−score	0.706	0.667	0.667	0.800	0.567	0.681
	Precision	0.792	0.913	**1.000**	**1.000**	**1.000**	**0.941**
	Recall	0.702	0.560	0.500	0.667	0.396	0.565
Unsupervised learning [15]	Time/s	2.18	2.21	1.97	3.09	2.54	2.40
	Acc/%	91.8	94.9	92.8	88.2	96.9	92.9
	F1−score	0.429	0.424	0.421	0.500	0.797	0.514
	Precision	0.614	0.683	0.308	0.400	0.671	0.535
	Recall	0.544	0.863	0.667	0.667	**0.979**	0.744
GraphSAGE [22]	Time/s	**1.52**	**1.51**	1.53	1.61	1.88	1.61
	Acc/%	94.4	91.2	85.0	88.2	96.2	91.0
	F1−score	0.667	0.400	0.080	0.500	0.758	0.481
	Precision	**1.000**	0.286	0.052	0.400	0.618	0.471
	Recall	0.500	0.667	0.167	0.667	**0.979**	0.596
EGAT [18]	Time/s	1.91	2.12	0.08	0.80	1.02	1.19
	Acc/%	86.3	93.8	92.0	68.8	70.8	82.3
	F1−score	0.724	0.826	0.600	0.546	0.340	0.607
	Precision	0.700	0.760	0.500	0.375	0.205	0.508
	Recall	0.750	0.905	0.750	**1.000**	**1.000**	0.881
MCE-HGCN	Time/s	1.68	1.81	**0.04**	**0.14**	**0.33**	**0.80**
	Acc/%	**96.3**	**97.1**	**99.4**	**99.3**	**98.8**	**98.2**
	F1−score	**0.874**	**0.920**	**0.923**	**0.957**	**0.913**	**0.917**
	Precision	0.787	0.851	0.857	**1.000**	0.855	0.870
	Recall	**1.000**	**1.000**	**1.000**	0.917	0.979	**0.979**

The best results for each metric in each case are highlighted in bold.

**Table 3:** Matching constraint extraction results with different optimization factors.

Metrics		130 nm OTAs	180 nm OTAs	130 nm LDOs	40 nm COMP	40 nm ADC	Average
MCE-HGCN without mixed attentions	Time/s	2.08	2.13	1.64	1.32	1.68	1.77
	Acc/%	94.1	94.7	94.8	83.5	89.7	91.4
	F1−score	0.560	0.583	0.200	0.743	0.726	0.542
	Precision	0.600	0.947	0.200	0.835	0.850	0.686
	Recall	0.533	0.421	0.200	0.310	0.633	0.419
MCE-HGCN without match filter	Time/s	1.82	1.90	0.05	0.84	1.09	1.14
	Acc/%	94.5	91.7	99.4	86.4	88.3	92.06
	F1−score	0.778	0.627	**0.923**	0.765	0.820	0.783
	Precision	**0.801**	0.700	**0.921**	0.749	0.850	0.804
	Recall	0.378	0.567	0.930	0.779	0.920	0.715
MCE-HGCN	Time/s	**1.68**	**1.81**	**0.04**	**0.14**	**0.33**	**0.80**
	Acc/%	**96.3**	**97.1**	**99.4**	**99.3**	**98.8**	**98.2**
	F1−score	**0.874**	**0.920**	**0.923**	**0.957**	**0.913**	**0.917**
	Precision	0.787	**0.851**	0.857	**1.000**	**0.855**	**0.868**
	Recall	**1.000**	**1.000**	**1.000**	**0.917**	**0.979**	**0.979**

The best results for each metric in each case are highlighted in bold.

## Data Availability

The original contributions presented in this study are included in the article. Further inquiries can be directed to the corresponding authors.
